# Supplementary material for: Comparison of 8- versus 12-weeks of glecaprevir/pibrentasvir for Taiwanese patients with hepatitis C and compensated cirrhosis in a real-world setting
Source: PLoS One. 2022 Aug 18;17(8):e0272567. doi: 10.1371/journal.pone.0272567 (PMC9387785; doi:10.1371/journal.pone.0272567)
Supplement: Attachment — Submitted filename: Response to Reviewers PONE-D-22-15455 R1.docx [file pone.0272567.s002.docx]

**Response to Reviewers (PONE-D-22-1545) R1**

**Comparison of 8- versus 12-weeks of glecaprevir/pibrentasvir for Taiwanese patients with hepatitis C and compensated cirrhosis in a real-world setting**

Yung-Hsin Lu^1^, Chung-Kuang Lu^1^, Chun-Hsien Chen^1^, Yung-Yu Hsieh^1^, Shui-Yi Tung^1,2^, Yi-Hsing Chen^1^, Chih-Wei Yen^1^, Wei-Lin Tung^1^, Kao-Chi Chang^1^, Wei-Ming Chen^1^, Sheng-Nan Lu^1,2,^, Chao-Hung Hung^1,2^, Te-Sheng Chang^1,2^*

^1^Division of Gastroenterology and Hepatology, Department of Internal Medicine, Chang Gung Memorial Hospital, Chiayi, Taiwan

^2^College of Medicine, Chang Gung University, Taoyuan, Taiwan

**Corresponding author:** Te-Sheng Chang, MD, PhD, No. 6, Section West, Chiapu Road, Puzi, Chiayi, 613, Taiwan, Tel: 886-5-3621000 Ext. 2005, Email: [cgmh3621@cgmh.org.tw](mailto:cgmh3621@cgmh.org.tw)

Dear Dr. Chang,

Thank you for submitting your manuscript to PLOS ONE. After careful consideration, we feel that it has merit but does not fully meet PLOS ONE’s publication criteria as it currently stands. Therefore, we invite you to submit a revised version of the manuscript that addresses the points raised during the review process.

==============================
COMMENTS FROM ACADEMIC EDITOR: The authors should pay more attention in making a more comprehensive review of the published articles, and avoid citing outdated and inadequate references. The content should be significantly reorganized before further consideration.

(1) The authors should discuss and compare the current knowledges with regard to first-line pan-genotypic DAAs in Taiwan, including GLE/PIB and SOF/VEL (Liu CH, et al Liver Int 2020, Huang CF et al. Sci Rep 2021; Liu CH, et al Hepatol Int 2021; Cheng PN, et a;. Infect Dis Ther 2022).

**Answer:** Discussion regarding the real-world experiences of the first-line pangenotypic DAAs, GLE/PIB and SOF/VEL, in Taiwan is added in the “Discussion”. (P12L6-8)

(2) Delete Reference No. 14 because cirrhosis is not a significant factor to predict SVR in the era of pan-GT DAAs, particularly for HCV GT1 and GT2. (If cirrhosis significantly impact SVR, why GLE/PIB to be shorten from 12 weeks to 8 weeks in compensated cirrhosis? As the authors stated in the study, all cirrhotic patients achieved SVR)

**Answer:** The authors agree that growing evidence has proven that cirrhosis is not a significant issue any more in predicting SVR for current new-generation pangenotypic DAAs. We have delete reference No. 14 as the editor suggested.

(3) Delete Reference No. 18 because it was only a phase II study. Please use Phase III trial GLE/PIB pooled analysis to support your discussion. (Puoti M, et al. J Hepatol 2018;69:293-300)

**Answer:** Thank you for kind reminder. The previous reference No. 18 is replaced by the updated phase III trial report (Puoti M, et al. J Hepatol 2018;69:293-300).

(4) Discussion: Strongly disagree with the illustration "Liver cirrhosis has long been considered a major factor in reducing the SVR rates of HCV treatment even in the era of DAA [14, 19]" Please delete the wordings in the Introduction and Discussion

**Answer:** The wordings specified by the editor in the “Introduction” and “Discussion” are deleted.

(5) In addition to Reference No., 23, 24. The authors should discuss three published articles (Klinker H, et al. Liver Int 2021;41:1518-22, Flamm SL, et al. Adv Ther 2020;37:2267-74, Zuckerman E, et al. Clin Gastroenterol Hepatol 2020;18:2544-53).

**Answer:** The three published articles specified by the editor are addressed and discussed in the “Discussion”. (P13L10-15, P13L22-25)

(6) Delete Reference No. 27 because it was a poorly written article.  Replace it with two articles (Liu CH, et al Liver Int 2020, Huang CF et al. Sci Rep 2021).

**Answer:** The previous reference No. 27 is replaced with the two articles specified by the editor.

(7) Discussion: Strongly disagree with the wording "This could also explain the lower eGFR in the 12-week group because GLE/PIB was one of the first TFDA-approved DAAs for patients with severe renal impairment, especially for those with genotype 2 HCV". Since this study comprised all genotypes, simply attributing to GT2 as the presence of significant difference in eGFR was not correct. The authors should stated a statistical difference for HCV GT2 patients in Results receiving 8 or 12-week GLE/PIB. The original wording should be replaced with "the first TFDA-approved DAAs for HCV GT2 patients with severe renal impairment"

**Answer:** The original sentence has been changed to the precise wording assigned by the editor.

(8) How about the potential drug-drug interactions (DDIs) in all these patients? Did these patients take "red contradictory co-medication" before or during GLE/PIB? Please also refer Taiwan DDI data (Liu CH, et al. Aliment Pharmacol Ther 2018) to show a higher risk of DDI compared to SOF-based DAAs, and to alert the healthcare providers to judiciously check DDI before and during GLE/PIB.

**Answer:** The importance regarding careful assessment for potential DDIs before prescribing the DAAs, especially for the GIE/PIB regimen is addressed in the “Discussion”. (P16L6-16)

==============================

Please submit your revised manuscript by Aug 11 2022 11:59PM. If you will need more time than this to complete your revisions, please reply to this message or contact the journal office at [plosone@plos.org](mailto:plosone@plos.org). When you're ready to submit your revision, log on to https://www.editorialmanager.com/pone/ and select the 'Submissions Needing Revision' folder to locate your manuscript file.

Please include the following items when submitting your revised manuscript:

- A rebuttal letter that responds to each point raised by the academic editor and reviewer(s). You should upload this letter as a separate file labeled 'Response to Reviewers'.
- A marked-up copy of your manuscript that highlights changes made to the original version. You should upload this as a separate file labeled 'Revised Manuscript with Track Changes'.
- An unmarked version of your revised paper without tracked changes. You should upload this as a separate file labeled 'Manuscript'.

If you would like to make changes to your financial disclosure, please include your updated statement in your cover letter. Guidelines for resubmitting your figure files are available below the reviewer comments at the end of this letter.

If applicable, we recommend that you deposit your laboratory protocols in protocols.io to enhance the reproducibility of your results. Protocols.io assigns your protocol its own identifier (DOI) so that it can be cited independently in the future. For instructions see: <https://journals.plos.org/plosone/s/submission-guidelines#loc-laboratory-protocols>. Additionally, PLOS ONE offers an option for publishing peer-reviewed Lab Protocol articles, which describe protocols hosted on protocols.io. Read more information on sharing protocols at <https://plos.org/protocols?utm_medium=editorial-email&utm_source=authorletters&utm_campaign=protocols>.

We look forward to receiving your revised manuscript.

Kind regards,

Chen-Hua Liu
Academic Editor
PLOS ONE

Journal Requirements:

When submitting your revision, we need you to address these additional requirements.

1. Please ensure that your manuscript meets PLOS ONE's style requirements, including those for file naming. The PLOS ONE style templates can be found at 
https://journals.plos.org/plosone/s/file?id=wjVg/PLOSOne_formatting_sample_main_body.pdf and 
<https://journals.plos.org/plosone/s/file?id=ba62/PLOSOne_formatting_sample_title_authors_affiliations.pdf>
**Answer:** The format of the manuscript has been modified to meet PLOS ONE’s style requirements.

2. Thank you for stating the following in the Acknowledgments Section of your manuscript:

"This work was supported by research grant CMRPG6J0173 from Chang Gung
Memorial Hospital."

We note that you have provided funding information that is not currently declared in your Funding Statement. However, funding information should not appear in the Acknowledgments section or other areas of your manuscript. We will only publish funding information present in the Funding Statement section of the online submission form. 
Please remove any funding-related text from the manuscript and let us know how you would like to update your Funding Statement. Currently, your Funding Statement reads as follows:

"This work was supported by research grant CMRPG6J0173 from Chang Gung Memorial Hospital to TSC.
The funder plays no role in the study design, data collection and analysis, decision to publish, or preparation of the manuscript"

Please include your amended statements within your cover letter; we will change the online submission form on your behalf.
**Answer:** The funding statement is deleted and all amended statements are included in the cover letter.
[Note: HTML markup is below. Please do not edit.]

Reviewers' comments:

Reviewer's Responses to Questions

**Comments to the Author**

1. Is the manuscript technically sound, and do the data support the conclusions?

The manuscript must describe a technically sound piece of scientific research with data that supports the conclusions. Experiments must have been conducted rigorously, with appropriate controls, replication, and sample sizes. The conclusions must be drawn appropriately based on the data presented.

Reviewer #1: Partly

Reviewer #2: Yes

2. Has the statistical analysis been performed appropriately and rigorously?

Reviewer #1: No

Reviewer #2: Yes

3. Have the authors made all data underlying the findings in their manuscript fully available?

The [PLOS Data policy](http://www.plosone.org/static/policies.action#sharing) requires authors to make all data underlying the findings described in their manuscript fully available without restriction, with rare exception (please refer to the Data Availability Statement in the manuscript PDF file). The data should be provided as part of the manuscript or its supporting information, or deposited to a public repository. For example, in addition to summary statistics, the data points behind means, medians and variance measures should be available. If there are restrictions on publicly sharing data—e.g. participant privacy or use of data from a third party—those must be specified.

Reviewer #1: Yes

Reviewer #2: Yes

4. Is the manuscript presented in an intelligible fashion and written in standard English?

PLOS ONE does not copyedit accepted manuscripts, so the language in submitted articles must be clear, correct, and unambiguous. Any typographical or grammatical errors should be corrected at revision, so please note any specific errors here.

Reviewer #1: Yes

Reviewer #2: Yes

5. Review Comments to the Author

Please use the space provided to explain your answers to the questions above. You may also include additional comments for the author, including concerns about dual publication, research ethics, or publication ethics. (Please upload your review as an attachment if it exceeds 20,000 characters)

Reviewer #1: It is an important mission to eliminate HCV by 2030. Therefore, effectiveness and safety of antiviral therapy are a critical issue. This real-world study pointed that the 8-week GP therapy were effectiveness as the 12-week GP therpay in patients with compensated liver cirrhosis. However, the novelty of this observative and descriptive study is relative low. There are some weak points to overcome.

[major problems]
1. The patients treated with the 8-week GP had higher platelet count, albumin level, Hb and eGFR values; lower proportion of prior HCC. It seemed that the patients receiving 8-week GP had less severe liver fibrosis/cirrhosis.
a). Please explain the reason(s)
**Answer:** We did explain the reasons in the “Discussion”: This phenomenon might also be explained by the timing of TFDA approval, because the 12-week GLE/PIB regimen was approved earlier than the 8-week regimen and patients who were aware of their liver disease generally had severer disease and were enrolled for treatment as soon as GLE/PIB was approved.(P15L3-9)
b). List Child-Pugh score (Table 1)
**Answer:** Child-Pugh score was added to Table 1 as the reviewer suggested.
c). Provide additional information (such as liver stiffness measurement or Fibrotest)
**Answer:** For the limitation of facility in routine clinical practice, Fibroscan and serum markers for the generation of Fibrotest including α2-macroglobulin, haptoglobin, apolipoprotein A1, and gamma glutamyl transpeptidase are not available.
2. The clinical data is not normal distribution. Please use median (Q1-Q3; Table 1).
**Answer:** The clinical data is expressed as median (interquartile range) instead of mean±SD as the reviewer suggested.
3. Please list the total and subgroup numbers of patients with FIB-4 >6 and FIB-4 between 3.25 and 6; please list detail numbers and percentage of the parameters for patients with a FIB-4 3.5-6.
**Answer:** The total and subgroup numbers of patients with FIB-4 >6 and FIB-4 3.25-6 are added in Table 1. The detailed numbers and percentage of the parameters for patients with FIB-4 of 3.25–6 are listed in the footnote of Table 1.
4. The difference of each AE between the 8- and 12-week GP therapy should be compared with P-value. (Table 3)
**Answer:** The *p*-value is provided in Table 3 as the reviewer suggested.
5. Any predictors of each/total AE? The predictors of clinical significant AE (such as ALT > 5 xULN) and direct hyperbilirubinemia (> 1.5 xUNL) should be investigated. (additional information is necessary)
**Answer:** Logistic regression is performed to investigate the predictors of clinically significant laboratory AEs. The results were listed below for your reference but I am not sure if it is necessary to put the table in the manuscript as the predictors for elevation of ALT and bilirubin seem to be of little or no clinical implication.

[minor problems]
1. It should be effectiveness "and safety" of GP for a treatment in patients with HCV and compensated cirrhosis. (line 12 & 14, page 4)
**Answer:** These statements have been amended as the reviewer suggested.
2. What dose superscript "6" mean? (line 3, page 5)
**Answer:** Thank you for kindly reminder. The superscript “6” is deleted.
3. Please give references to support "...this AE (pruritis) can increase with longer treatment duration (line 7, page12)."
**Answer:** There is no any literature reporting an increased rate of pruritus with longer treatment duration by GLE/PIB. After statistical analysis, there is no significant difference in the rates of pruritus between the two groups and the statement regarding increased rate of pruritus with longer treatment duration by GLE/PIB is removed.
4. Indirect hyperbilirubinemia is less clinically significant. Please list total bilirubin > 1.5 x or 3 x ULN AND direct hyperbilirubinemia. (Table 3)
**Answer:** Rate of direct hyperbilirubinemia is listed in Table 3 as the reviewer suggested.
5. Footnotes of Table 1 and 3 were missed.
**Answer:** Footnotes of Table 1 and 3 are added.
6. The dataset in the Supporting Information was Chinese records.
**Answer:** The Chinese characters in the dataset are amended and the birthdates are deleted to avoid release of personal identity.

Reviewer #2: This study aimed to compare the treatment efficacy and safety between 8-week and 12-week GLE/PIB therapy for patients with compensated HCV-related liver cirrhosis. The authors showed that both 8-week and 12-week GLE/PIB therapy provide excellent antiviral efficacy and safety profiles. Although it is interesting and has clinical implications, several concerns need to be clarified.
1. In this study, 7 patients had HBV and HCV dual infections. Were they treated with antiviral therapy for HBV? How many patients developed HBV reactivation during GIE/PIB therapy for HCV infection?
**Answer:** There were 13 patients with dual HBV and HCV infections, seven in the 8-week group and six in the 12-week group. Among them, 4 patients were undergoing antiviral therapy for HBV, one in the 8-week group (treated with entecavir) and three in the 12-week group (two with entecavir and one with tenofovir alafenamide). No HBV reactivation was observed during GIE/PIB therapy for these 13 patients. These descriptions are added in the “Discussion”.(P15L23-P16L5)
2. Moreover, 5 patients had a history of HCC. Were they active or non-active during GLE/PIB therapy? Did they undergo therapy for HCC during DAA therapy?
**Answer:** Four of the 5 patients in the 8-week group had active hepatocellular carcinoma (HCC), one received transcatheter arterial chemoembolization and three received radiofrequency ablation during GLE/PIB therapy. Four of the 16 patients in the 8-week group had active HCC, none received treatment for HCC during GLE/PIB therapy. These descriptions are added in the footnote of Table 1.
3. How many patients had clinically significant portal hypertension (CSPH)? Were the rates of CSPH different between these two groups of patients?
**Answer:** Since this study is a retrospective analysis, there were no reliable measurements fitting the definition of clinically significant portal hypertension (CSPH). However, the number of patients with CSPH is expected to be extremely low as only patients with compensated cirrhosis were included.
4. Were the rates of biochemical response or ALT normalization different between these two groups of patients?
**Answer:** There was no difference regarding the rates of biochemical response and the rates and comparisons between the two groups are added in Table 2.
5. In this study, patients in the 12-week treatment group had a lower baseline eGFR than those in the 8-week treatment group. Were the dynamic changes of eGFR during and after DAA therapy different between these two groups of patients?
**Answer:** The 12-week treatment group had lower dynamic changes of eGFR compared to the 8-week treatment group (*p* = 0.0003). This result was expressed as Figure 3 and addressed in the “Discussion”.(P13L11-13)
6. It is suggested to provide a table to compare the laboratory data (such as albumin, bilirubin, ALT, platelet count, FIB-4, eGFR, etc.) at the time of end of follow-up (SVR12) between 8-week and 12-week treatment groups.
**Answer:** These comparisons are provided as below for your reference. The laboratory parameters with *p* < 0.05 at SVR12 between the two groups were the same as those at baseline, including hemoglobin, platelet count, albumin, creatinine and eGFR as shown in Table 1.

| SVR12 lab data | 8 Weeks  (N=91) | 12 Weeks  (N=68) | *p* value |
| --- | --- | --- | --- |
| FIB-4, median (IQR) | 3.6 (2.8-4.2) | 3.6 (2.9-4.6) | 0.35 |
| White blood cell count, 10^9^ cells/L, median (IQR) | 5.4 (4.5-6.6) | 5.2 (4.3-7) | 0.7 |
| Hemoglobin, g/dL, median (IQR) | 13.2 (12-14.4) | 12.4 (11.1-14) | 0.03* |
| Platelet count, 10^9^ cells/L, median (IQR) | 131 (107.5-151.5) | 112 (93-139) | 0.02* |
| Albumin, g/dL, median (IQR) | 4.2 (4.1-4.4) | 4 (3.8-4.3) | 0.001* |
| Total bilirubin, mg/dL, median (IQR) | 0.8 (0.6-1.1) | 0.8 (0.7-1.1) | 0.54 |
| AST, U/L, median (IQR) | 27 (21-36.5) | 27 (21-33) | 0.57 |
| ALT, U/L, median (IQR) | 20 (14-28.5) | 20 (16-31) | 0.62 |
| Creatinine, mg/dL, median (IQR) | 0.9 (0.8-1.1) | 1 (0.8-2.1) | 0.007* |
| eGFR, mL/min/1.73m^2^, median (IQR) | 74.4 (64.2-86.1) | 64 (33.1-84.5) | 0.02* |
| Alpha-fetoprotein, ng/mL, median (IQR) | 3.6 (2.4-5.4) | 3.5 (2.6-5.6) | 0.78 |

7. In Table 1, it is suggested to provide the exact p value instead of p< 0.05.
**Answer:** The exact *p* values are provided in Table 1.

6. PLOS authors have the option to publish the peer review history of their article ([what does this mean?](https://journals.plos.org/plosone/s/editorial-and-peer-review-process#loc-peer-review-history)). If published, this will include your full peer review and any attached files.


If you choose “no”, your identity will remain anonymous but your review may still be made public.


**Do you want your identity to be public for this peer review?** For information about this choice, including consent withdrawal, please see our [Privacy Policy](https://www.plos.org/privacy-policy).

Reviewer #1: No

Reviewer #2: No

[NOTE: If reviewer comments were submitted as an attachment file, they will be attached to this email and accessible via the submission site. Please log into your account, locate the manuscript record, and check for the action link "View Attachments". If this link does not appear, there are no attachment files.]

While revising your submission, please upload your figure files to the Preflight Analysis and Conversion Engine (PACE) digital diagnostic tool, <https://pacev2.apexcovantage.com/>. PACE helps ensure that figures meet PLOS requirements. To use PACE, you must first register as a user. Registration is free. Then, login and navigate to the UPLOAD tab, where you will find detailed instructions on how to use the tool. If you encounter any issues or have any questions when using PACE, please email PLOS at [figures@plos.org](mailto:figures@plos.org). Please note that Supporting Information files do not need this step.
